# The blues, and an almost shocking surprise – Unexpected PE in a catatonic patient, that almost had ECT

**DOI:** 10.1192/bjo.2021.373

**Published:** 2021-06-18

**Authors:** Michael Cheah, Ashma Mohamed, Anand Mathilakath

**Affiliations:** Surrey and Borders Partnership NHS Foundation Trust

## Abstract

**Aims:**

To present a case of a near-miss, where an unexpected Pulmonary Embolism (PE) was identified in a patient with psychotic depression and catatonia, who almost had Electroconvulsive Therapy (ECT). Our aim is to highlight the importance of Venous-Thrombo Embolism (VTE) risk assessment in all psychiatric inpatients, particularly those with catatonia, and those about to undergo ECT.

**Method:**

A 53-year-old female admitted with her first presentation of psychotic depression, catatonia, poor oral intake, and significant weight loss in the community for months prior to admission. She was recommended for emergency ECT as the severity of her self-neglect was becoming life threatening. Her first ECT session was cancelled due to low potassium levels prior to ECT, which proved to be a fortunate event. She developed sudden onset chest pain the next day, and following further medical investigations; was diagnosed to have a bilateral PE, and subsequently treated with Apixaban. Due to the potential risk of ECT dislodging the clots, treatment was done by optimising medication alone; Venlafaxine 300 mg, Mirtazapine 45 mg, Haloperidol 6 mg. She made a slow but successful recovery, and was discharged home, with ongoing support from Early Intervention in Psychosis services.

**Result:**

We conducted a literature search, and it is well known that there is an increased risk of VTE in catatonic patients, as well as other psychiatric inpatients; due to anti-psychotic medication. Furthermore, cases have been reported where ECT was associated with increased risk of death in patients with known VTE/PE.

On retrospective review of the patient's risks of developing VTE in the community, it was clear, that she was at very high risk of developing VTE. It was also noted that she should have had a VTE risk assessment on admission, in accordance with NICE guidelines; where all acute psychiatric inpatients should have this assessed as soon as possible.

**Conclusion:**

Through a process of assessment and treatment, VTE is often preventable. Identification of high-risk patients on admission to hospital is therefore crucial. It is thus, imperative that a comprehensive VTE risk assessment is completed on admission and regularly reviewed.

This case highlights the risk of missing VTE assessments in WAA Inpatients, particularly those with catatonia, about to undergo ECT, which could have been fatal. As such, VTE/PE risk assessment in such patients, about to undergo ECT, is particularly crucial.

Clinicians need to have a high index of suspicion of VTE/PE, particularly in patients with catatonia.

